# Mitotic count can predict tamoxifen benefit in postmenopausal breast cancer patients while Ki67 score cannot

**DOI:** 10.1186/s12885-018-4516-1

**Published:** 2018-07-24

**Authors:** Karin Beelen, Mark Opdam, Tesa Severson, Rutger Koornstra, Andrew Vincent, Jelle Wesseling, Joyce Sanders, Jan Vermorken, Paul van Diest, Sabine Linn

**Affiliations:** 1 Molecular Biology, The Netherlands Cancer Institute, Amsterdam, The Netherlands; 2 Departments of Biometrics, The Netherlands Cancer Institute, Amsterdam, The Netherlands; 3 Pathology, The Netherlands Cancer Institute, Amsterdam, The Netherlands; 4 Department of Medical Oncology, University Hospital Antwerpen, Edegem, Belgium; 5 Department of Pathology, University Medical Center Utrecht, Utrecht, The Netherlands; 6 Medical Oncology, The Netherlands Cancer Institute, Plesmanlaan 121, 1066 CX Amsterdam, The Netherlands

**Keywords:** Breast cancer, Tamoxifen, Cell proliferation, Ki67, Mitotic count

## Abstract

**Background:**

Controversy exists for the use of Ki67 protein expression as a predictive marker to select patients who do or do not derive benefit from adjuvant endocrine therapy. Whether other proliferation markers, like Cyclin D1, and mitotic count can also be used to identify those estrogen receptor α (ERα) positive breast cancer patients that derive benefit from tamoxifen is not well established. We tested the predictive value of these markers for tamoxifen benefit in ERα positive postmenopausal breast cancer patients.

**Methods:**

We collected primary tumor blocks from 563 ERα positive patients who had been randomized between tamoxifen (1 to 3 years) vs. no adjuvant therapy (IKA trial) with a median follow-up of 7.8 years. Mitotic count, Ki67 and Cyclin D1 protein expression were centrally assessed by immunohistochemistry on tissue microarrays. In addition, we tested the predictive value of *CCND1* gene copy number variation using MLPA technology. Multivariate Cox proportional hazard models including interaction between marker and treatment were used to test the predictive value of these markers.

**Results:**

Patients with high Ki67 (≥5%) as well as low (< 5%) expressing tumors equally benefitted from adjuvant tamoxifen (adjusted hazard ratio (HR) 0.5 for both groups)(p for interaction 0.97). We did not observe a significant interaction between either Cyclin D1 or Ki67 and tamoxifen, indicating that the relative benefit from tamoxifen was not dependent on the level of these markers. Patients with tumors with low mitotic count derived substantial benefit from tamoxifen (adjusted HR 0.24, *p* <  0.0001), while patients with tumors with high mitotic count derived no significant benefit (adjusted HR 0.64, *p* = 0.14) (p for interaction 0.03).

**Conclusion:**

Postmenopausal breast cancer patients with high Ki67 counts do significantly benefit from adjuvant tamoxifen, while those with high mitotic count do not. Mitotic count is a better selection marker for reduced tamoxifen benefit than Ki67.

**Electronic supplementary material:**

The online version of this article (10.1186/s12885-018-4516-1) contains supplementary material, which is available to authorized users.

## Background

Decisions on adjuvant systemic therapy in breast cancer are generally made on the basis of clinico-pathological variables that may predict both prognosis and treatment efficacy. While tumor size, lymph node status and histological grade are important factors to predict prognosis (and to decide whom to treat), hormone receptor status and HER2 status can predict both prognosis and treatment efficacy for respectively endocrine treatment and HER2 blockade. Low hormone receptor levels have been associated with reduced efficacy of endocrine therapy [1] and increased benefit from cytotoxic agents [2, 3] compared to higher levels. Contrary to the predictive value of hormone receptor, the predictive value of Ki67 labeling index for benefit from endocrine therapy is less clear [4–7]. In the NSABP B-14 trial, comparing adjuvant tamoxifen with placebo, proliferation genes like Ki67 did not significantly interact with treatment [8]. Retrospective analysis of Ki67 in a randomized trial in premenopausal patients, identified a complex relation between Ki67 and benefit from tamoxifen; patients whose tumors expresses high or low Ki67 expression benefitted more from tamoxifen compared to patients whose tumor expressed intermediate levels of Ki67 [5]. No predictive role for benefit of chemotherapy over endocrine therapy alone has been shown for patients with high tumor Ki67 expression [9]. A weak association between high Ki67 levels and increased benefit from aromatase inhibition over tamoxifen was observed in the BIG 1–98 trial [6].

The efficacy of adjuvant endocrine therapy may also be affected by proliferation markers other than Ki67. An example is Cyclin D1, which is involved in G1 progression. In addition to its role in cell cycle progression, Cyclin D1 can also enhance ligand independent activation of ERα [10]. The sensitivity of tumor cells with high Cyclin D1 expression to selective estrogen receptor modulators has been found to be compound specific, but no effect on the in vitro efficacy of tamoxifen was shown [11, 12]. Also clinically, Cyclin D1 protein expression was not associated with efficacy of tamoxifen in premenopausal patients randomized between either tamoxifen or control [13]. The gene encoding Cyclin D1, *CCND1*, is located in a frequently amplified region, 11q13 [14]. In premenopausal patients randomized to tamoxifen versus control, the efficacy of tamoxifen was reduced in patients whose tumor carried *CCND1* gene amplification as defined with FISH [15]. In postmenopausal patients, however, amplification of *CCND1,* as defined with realtime-PCR, did not have independent predictive value [16]. In this series, amplification of a gene in the same region, *PAK1* (also known to affect the ERα) did actually reduce tamoxifen efficacy [16].

A proliferation marker that is assessed as a standard clinico-pathological variable is the mitotic count, the main factor contributing to the modified Bloom-Richardson grading score [17]. Although mitotic count is clearly associated with breast cancer prognosis [18], it is unclear whether the mitotic count affects the efficacy of endocrine therapy.

The aim of our study was therefore to determine the predictive value of Ki67 protein expression and other proliferation markers for efficacy of tamoxifen in postmenopausal breast cancer patients randomized to tamoxifen versus no systemic treatment. The clinical decision to omit adjuvant chemotherapy and only advise adjuvant tamoxifen could be strengthened in case low proliferation as measured with one or more of the examined markers is associated with substantial tamoxifen benefit. This could especially be of added benefit when multigene assays return equivocal results regarding this issue, such as an intermediate-risk 21-gene recurrence score [19].

## Methods

### Patients and material

We have recollected tissue blocks with sufficient tumor material of 739 patients who participated in a Dutch randomized clinical trial, studying the benefit of adjuvant tamoxifen in postmenopausal breast cancer patients (IKA-trial). The patient characteristics and clinical outcome of tamoxifen treatment of the original study group (1662 patients) have been presented elsewhere [20] and were part of the Oxford meta-analysis [21]. The numbers of patients in each treatment arm pre- and post-interim analysis have been presented previously [22]. Prognostic factors in these 739 patients did not differ from the total group (Additional file 1: Table S1). After revision of estrogen receptor α (ERα) status as assessed with immunohistochemistry, a total of 563 ERα positive tumors were used for subsequent analysis. Median follow-up of patients without a recurrence event is 7.8 years. When stratified by nodal status, the adjusted hazard ratio regarding recurrence-free interval for tamoxifen versus control in ERα positive patients is 0.54 (95% CI 0.36–0.83, *p* = 0.004).

### Immunohistochemistry

Tissue microarrays (TMAs) were constructed using formalin-fixed paraffin embedded (FFPE) tumor blocks. A total of three (0.6 mm) cores per tumor were embedded in the TMAs that were stained for ERα, progesterone receptor (PgR) and HER2 as described previously [23]. Tumor grade was scored on hematoxylin-eosin (HE) stained slides using the modified Bloom-Richardson score [17]. The mitotic count was assessed (PvD) per 2 mm^2^ as before [24].

Immunohistochemistry for Ki67 was performed using the monoclonal mouse anti-human Ki67 antigen, clone MIB-1 (DAKO, Agilent Technologies, Santa Clara, California, USA) and a standard staining protocol on the Ventana Benchmark® Ultra system (Ventana Medical Systems, Tucson, USA). Cyclin D1 protein expression was assessed using the Cyclin D1/ Bcl-1(SP4) antibody (Neomarkers, Portsmouth, USA) and a standard staining protocol on the Labvision system (Thermo Fisher Scientific Inc., Waltham, USA). For both stainings, the proportion of invasive tumor cells with nuclear staining was assessed by the first observer (MO). For each staining, one of the TMAs was quantified independently in a blinded manner by a second observer (JS) to calculate inter-observer variability. The inter-observer variability was analyzed using the Cohen’s kappa coefficient, which is depicted in Additional file 1 Table S2. The maximum score of the 3 cores as assessed by the first observer was used for analysis. For a random series of 55 tumors, whole tissue slides were stained and scored by one observer (MO) for Ki67 and results were compared with TMA scores.

### DNA isolation

DNA isolation was performed as previously described [25].

### *CCND1* gene copy number variation

*CCND1* gene copy number variation was assessed with multiplex ligation-dependent probe amplification based copy number analysis (MLPA). The P078-B1 Breast tumor probe-mix (MRC Holland, Amsterdam, The Netherlands) was used, which contains probe sets for several genes that frequently show copy number changes in breast tumors. The probe mix contains 2 different targeted probe sets for *CCND1*, one at chr11: 69465909–69,465,963 and the other at chr11: 69458599–69,458,665 (hg19). It also contains 2 probe sets for *EMSY*, a gene which is also located in the 11q13 region, closer to *PAK1*. Fig. S1 shows the location of the different *CCND1* and *EMSY* probe sets in the genome. The probe mix additionally contains 11 reference probe sets. We carried out MLPA reactions according to the manufacturer’s protocols for 2010 (see Appendix). For normalization of the signals, we discarded 5 references probe sets that exhibited high between batch variation (Additional file 2: Figure S2). The log2 transformed signal of each *CCND1* and *EMSY* probe set was normalized by dividing by the sum of the log2 transformed signal of the 6 remaining reference probe sets. Similarly, the log2 transformed signal of each reference DNA sample was normalized by dividing by the sum of the log2 transformed signal of the 6 remaining reference probe sets. For each gene, the ratio between the normalized signal of each patient sample and the mean normalized signal of the reference DNA, was subsequently used for data-analysis (and will be referred to as log2 copy number ratio).

### Statistical methods

Recurrence free interval was defined as the time from the date of first randomization until the occurrence of a local, regional or distant recurrence or breast cancer specific death. Since a secondary contra-lateral breast tumor cannot be inferred from the characteristics of the primary tumor, while the other type of events can in relation to tamoxifen resistance, this was not considered as an event and these patients were censored at the date of this occurrence. To test whether the benefit from tamoxifen treatment was dependent on proliferation markers, unadjusted and co-variable adjusted Cox proportional hazard regressions were performed including treatment-by-biomarker interaction tests. Treatment groups were defined according to the results of the first randomization (1–3 years of tamoxifen versus no adjuvant systemic treatment). The change in randomization that occurred after the interim analysis resulted in an enrichment of lymph node positive patients in the group of tamoxifen treated patients. Therefore, Cox proportional hazard regression models were stratified for nodal status. Continuous linear variables were tested: Ki67 score, mitotic count (square root transformed), Cyclin D1and *CCND1* and *EMSY* log2 copy number ratio (probe sets 1 and probe sets 2 were tested separately). In addition, we tested Ki67, mitotic count and Cyclin D1 as binary factors using the median as cutoff. For analysis of *CCND1* and *EMSY* log2 copy number ratio as binary factor, 0 was defined as cutoff. For all tested variables, proportional hazard assumption was tested and in case of a failure of proportional hazards, the interaction was tested separately for a time period without failure of proportional hazards, as indicated by Schoenfeld residuals. Co-variables included age (≥ 65 versus < 65), grade (grade 3 versus grade 1–2), tumor size (T3–4 versus T1-T2), HER2 status (positive versus negative), and PgR status (positive versus negative). We did not adjust for multiple testing. Survival curves were constructed using the Kaplan Meier method and compared using the log-rank test. This study complied with reporting recommendations for tumor marker prognostic studies (REMARK) criteria [26] as outlined in Additional file 1: Table S3.

## Results

### Success rate of cell cycle marker assessment

Mitotic count could adequately be assessed in 557/563 (99%) of ERα positive tumors. Immunohistochemistry for Cyclin D1 and Ki67 on TMA was successful in 442 and 423 tumors, respectively (Additional file 2: Figure S3). We did not observe a significant difference between Ki67 scores on whole slides compared to TMA scores (*p* = 0.38) (Additional file 2: Figure S4). Analyses of inter-observer variability for Ki67 and Cyclin D1 resulted in kappa values of 0.89 and 0.55, respectively (Additional file 1: Table S2).

Sufficient DNA for MLPA was available for 494/563 tumors. *CCND1* gene copy number variation could be assessed in 486 (98%) tumors for probe set 1 and 476 (96%) tumors for probe set 2. *EMSY* gene copy number variation could be assessed in 491 (99%) tumors for probe set 1 and 492 (99%) tumors for probe set 2 (Additional file 2: Figure S3). The distribution of the scores for the different cell cycle markers is depicted in Additional file 2: Figure S5.

### Mitotic count, Ki67 and differential benefit from tamoxifen

**Fig. 1:**
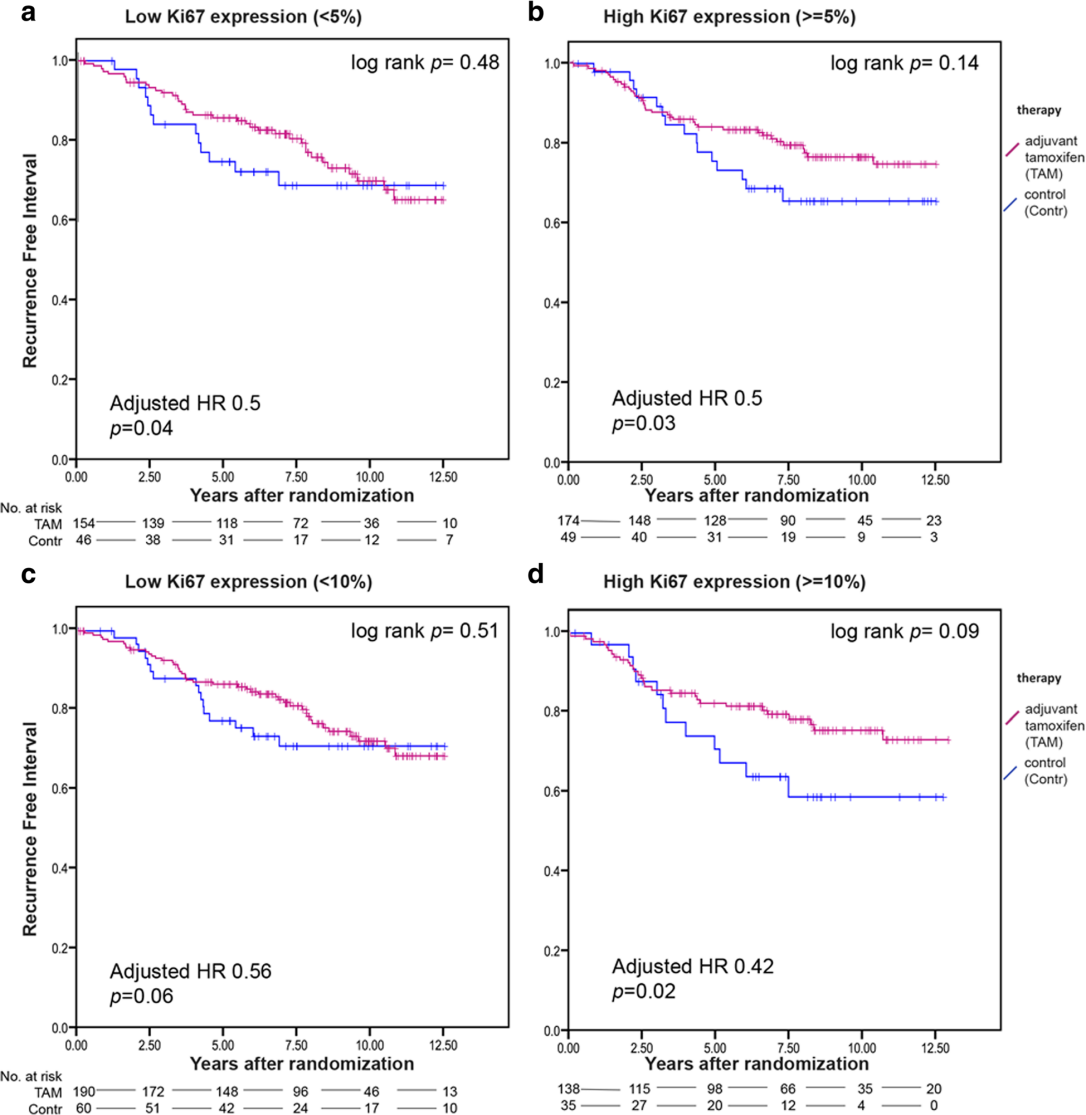
Kaplan Meier survival analyses for recurrence-free interval according to tamoxifen treatment in patients with a tumor with low or high Ki67 expression (cut-off at 5% expression level) (**a**, **b**) or low and high Ki67 expression (cut-off at 10% expression level) (**c**, **d**). The treatment-by-biomarker p interaction is 0.97 (5% cut off), or 0.52 (10% cut off)

**Fig. 2:**
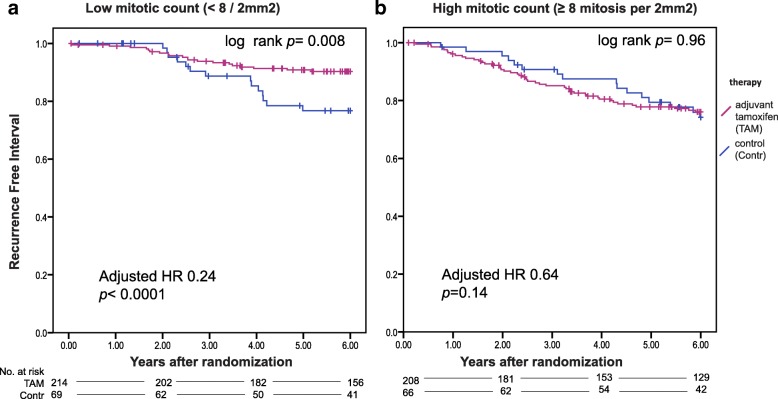
Kaplan Meier survival analyses (truncated at 6 years) for recurrence-free interval according to tamoxifen treatment in patients with a tumor with low mitotic count (**a**) and high mitotic count (**b**)

We did not find a significant interaction between treatment and the expression of Ki67 (Tables 1, 2 and Fig. 1). Patients with high Ki67 count (defined as > = 5% expression (Fig. 1a, b) or > =10% expression (Fig. 1c,d) did significantly benefit from adjuvant tamoxifen. For the mitotic count, analyzing the total follow up, no significant interaction with treatment was found. However, evidence of a failure of proportional hazards was observed (*p* = 0.07) in the univariate Cox-model for mitotic count. Schoenfeld residuals (Additional file 2: Figure S6) suggested a change in effect around 6 years. Tumors with high mitotic count were more likely to relapse than those with low mitotic count in the first 6 years. However, after 6 years, risks for recurrence were comparable. In a survival analysis in which follow-up was truncated at 6 years, we observed a significant interaction between treatment and mitotic count, analyzed as binary factor (*p* = 0.03). Patients with a tumor with low mitotic count (< 8 mitotic per 2mm^2^) derived substantial and significant benefit from tamoxifen (adjusted HR 0.24, 95% confidence interval 0.12–0.49, *p* <  0.0001), while patients with a tumor with high mitotic count (≥ 8 mitotic per 2mm^2^) did not (adjusted HR 0.64, 95% confidence interval 0.35–1.17, *p* = 0.14) (Fig. 2 and Tables 1, 2 and Additional file 1: Table S4).

**Table 1:** Covariate adjusted interaction tests between tamoxifen treatment efficacy according to recurrence-free interval and different cell proliferation markers analyzed as continuous linear variables. Co-variables included age (≥ 65 versus < 65), grade (grade 3 versus grade 1–2), tumor size (T3–4 versus T1-T2), HER2 status (positive versus negative), and PgR status (positive versus negative)

			Total follow-up	Follow-up truncated at 6 years^a^	
Variable	Variable values	*N* (events)	*p*-value	*N* (events)	*p*-value
Mitotic count square root	0 to 11	515 (124)	0.16	515 (92)	0.09
Cyclin D1 (continuous)	0 to 100%	432 (105)	0.96		
Ki67 (continuous)	0 to 100	407 (99)	0.30		
*CCND1* probeset 1 log 2copy number ratio	−2.43 to 3.36	450 (103)	0.21		
*CCND1* probeset 2 log2 copy number ratio	−3.46 to 4.00	439 (102)	0.002		

^a^Analysis performed for mitotic count only, since failure of proportional hazard assumption was observed

**Table 2:** Covariate adjusted interaction tests between tamoxifen treatment efficacy according to recurrence-free interval and different cell proliferation markers analyzed as binary factor. Co-variables included age (≥ 65 versus < 65), grade (grade 3 versus grade 1–2), tumor size (T3–4 versus T1-T2), HER2 status (positive versus negative), and PgR status (positive versus negative)

Variable	levels	HR (95% CI) for tamoxifen vs control (total follow-up)	Interaction *p*-value	HR (95% CI) for tamoxifen vs control (follow-up truncated at 6 years^a^)	Interaction *p-*value
Mitotic count	< 8/ 2mm^2^	0.38 (0.20–0.70)	0.13	0.24 (0.12–0.49)	0.03
	≥ 8/ 2mm^2^	0.70 (0.40–1.23)		0.64 (0.35–1.17)	
Cyclin D1	≤ 50%	0.48(0.25–0.93)	0.48		
	> 50%	0.66 (0.34–1.29)			
Ki67	< 5%	0.50 (0.26–0.98)	0.97		
	≥ 5%	0.50 (0.26–0.93)			
*CCND1* probeset 1 log 2copy number ratio	< 0	0.39 (0.18–0.86)	0.33		
	> 0	0.62 (0.18–1.07)			
*CCND1* probeset 2 log2 copy number ratio	< 0	0.32 (0.16–0.61)	0.04		
	> 0	0.81 (0.44–1.52)			

^a^Analysis performed for mitotic count only, since failure of proportional hazard assumption was observed

Analyzing HER2 negative patients only did not substantially change these results (interaction between tamoxifen and mitotic count *p* = 0.07) (Additional file 1: Table S5). We had insufficient power to analyze these differences separately in patients whose tumor had either low or high Ki67 expression.

High mitotic count was significantly associated with poor prognostic features like positive lymph node status, T stage as well as negative PgR status and positive HER2 status. In addition, we found significant associations between mitotic count and other cell proliferation markers like Ki67 and Cyclin D1 protein expression (Table 3).

**Table 3:** Association between mitotic count (left columns) and *CCND1* (right columns) and clinico-pathological variables and other cell proliferation markers

Variable	levels	Mitotic count per 2 mm2			CCND1 copy number ratio^b^		
		< 8 / 2 mm2	≥ 8 / 2 mm2		< 0	> 0	
		*N (%)*	*N (%)*	*p* value^a^	*N (%)*	*N (%)*	*p* value^a^
Age	< 65	133 (47)	133 (49)	0.72	101 (49)	129 (48)	0.72
	≥ 65	150 (53)	141 (51)		104 (51)	142 (52)	
Nodal status	negative	173 (61)	135 (49)	0.005	112 (55)	152 (56)	0.75
	positive	110 (39)	139 (51)		93 (45)	119 (44)	
T stage	T1–2	262 (93)	235 (86)	0.01	180 (88)	245 (90)	0.36
	T3–4	21 (7)	39 (14)		25 (12)	26 (10)	
Grade	grade 1–2	279 (99)	90 (33)	< 0.001	142 (69)	171 (63)	0.16
	grade 3	4 (1)	184 (67)		63 (31)	100 (37)	
PgR	negative	116 (42)	143 (52)	0.02	92 (46)	132 (50)	0.42
	positive	157 (58)	130 (48)		108 (54)	133 (50)	
Her2	negative	257 (93)	226 (84)	< 0.001	180 (90)	233 (87)	0.46
	positive	3 (1)	38 (14)		13 (7)	22 (8)	
	missing	16 (6)	6 (2)		7 (4)	12 (4)	
Ki67	< 5%	104(53)	94 (42)	0.02	72 (54)	92 (46)	0.78
	≥ 5%	92 (47)	130 (58)		88 (55)	106 (54)	
Cyclin D1	below median	106 (53)	100 (42)	0.02	76 (49)	105 (48)	0.85
	above median	93 (47)	140 (58)		80 (51)	115 (52)	
mitotic count	< 8 per 2 mm2	283 (100)	0(0)	na	116 (57)	126 (47)	0.03
	≥ 8 per 2 mm2	0(0)	274 (100)		87 (43)	142 (53)	
*CCND1* log 2 copy number ratio^b^	< 0	116 (48)	87 (38)	0.03	205 (100)	0(0)	na
	> 0	126 (52)	142 (62)		0(0)	271 (100)	

^a^Chi-square test, analysis based on cases without missing values

^b^probe set 2

### High *CCND1* copy number ratio is associated with tamoxifen resistance

**Fig. 3:**
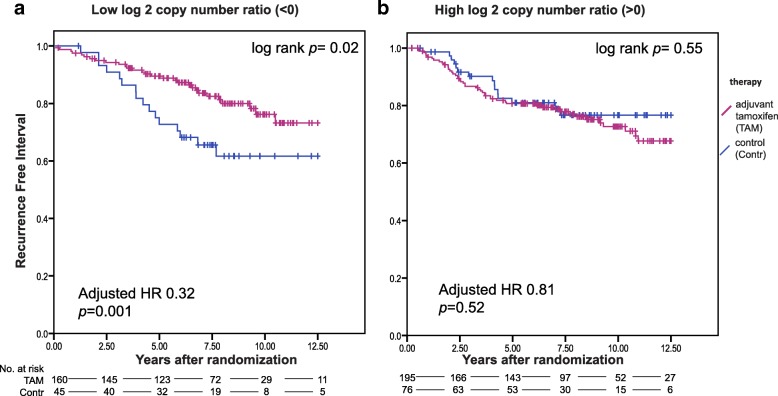
Kaplan Meier survival analyses according to tamoxifen treatment in patients with a tumor with low *CCND1* log2 copy number ratio (**a**) and high log2 copy number ratio (**b**)

We did not find a significant interaction between tamoxifen treatment and the expression of Cyclin D1, indicating that the efficacy of tamoxifen is not significantly different between patients whose tumor express low Cyclin D1 and patients whose tumor express high levels of Cyclin D1. For *CCND1*, we observed no interaction between probe set 1 and treatment (Tables 1 and 2). However, for the second probe set we observed a significant interaction with treatment both in the unadjusted as well as the adjusted analysis (*p* = 0.005 and 0.002 respectively). Patients whose tumor had higher *CCND1* log2 copy number ratio derived no significant benefit from tamoxifen. When analyzed as binary factor, patients with a log2 copy number ratio of less than 0 derived substantial and significant benefit from tamoxifen (adjusted HR 0.32, 95% confidence interval 0.16–0.61, *p* = 0.001) while those patients with a *CCND1* log2 copy number ratio above 0 did not (adjusted HR 0.81, 95% confidence interval 0.44–1.52, *p* = 0.52) (Fig. 3 and Tables 1, 2 and Additional file 1: Table S6).

Although patients with high *CCND1* log2 copy number ratio had more often tumors with high mitotic count (*p* = 0.03), we did not observe significant associations between *CCND1* log2 copy number ratio and other cell cycle markers (Table 3).

**Fig. 4:**
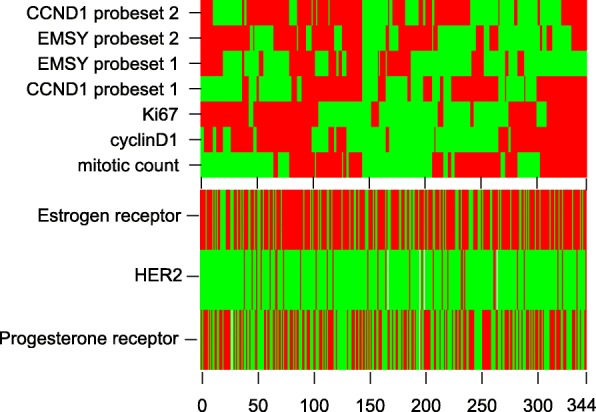
Heat map representing unsupervised hierarchical clustering of tumor samples and corresponding cell cycle markers and EMSY data. Patients are represented horizontally. Cell cycle markers and EMSY data are indicated vertically. Red represents marker expression above median and green represents expression below median. In addition, the status of ERα (100% (red) or below 100% (green)), PR (present (red) or absent (green)) and HER2 overexpression (present (red) or absent (green)) is shown

To explore co-amplification of other regions in the 11q13 region that may possibly cause tamoxifen resistance we analyzed the association between *CCND1* and *EMSY* log2 copy number ratio. We found a significant, albeit weak association between the second *CCND1* probe set and the second *EMSY* probe set, but not between the other probe sets (data not shown). None of the *EMSY* probe sets by itself was significantly associated with a difference in benefit from tamoxifen (Additional file 1: Table S7). Figure 4 shows a heat map of unsupervised hierarchical clustering of all analyzed cell cycle markers as well as the EMSY probesets.

## Discussion

Although cell proliferation markers are generally used to predict prognosis and are, together with hormone receptors, a major component of several clinically used prognostic multigene assays [27, 8] the ability of these markers to predict benefit from endocrine therapy has not well been established. We here show that in patients whose tumors express high mitotic count, tamoxifen efficacy is reduced.

In our series we did not observe an association between Ki67 labeling and tamoxifen efficacy. Nevertheless, Ki67 labeling is recommended as a standard variable to determine surrogate definitions of the intrinsic subtypes, enabling to predict prognosis and decide on optimal adjuvant systemic therapy. According to the St. Gallen guidelines, patients with low Ki67 expression would have been recommended adjuvant endocrine therapy only. In our series, in approximately half of the patients with low Ki67 expression, the mitotic count was above the threshold that predicted reduced tamoxifen efficacy. This implies that mitotic count outperforms Ki67 in prediction of the likelihood of deriving benefit from endocrine therapy alone. As expected, almost all tumors with histological grade III had a mitotic count > = 8/mm2. Most current guidelines recommend the addition of chemotherapy to endocrine therapy in grade III tumors. The clinical added value of mitotic count might therefore lay in the subgroup of histological grade I/II tumors. Of these, 24% (90 out of the 369) had a high mitotic count and might be considered for adjuvant chemotherapy in addition to endocrine therapy.

One explanation for the relatively low expression of Ki67 in our series is that the patient population was ERα positive and postmenopausal, reflecting a subset of patients with relatively low proliferating tumors. Although in the past there have been concerns about the reliability of Ki67 on TMAs, recently another study demonstrated that Ki67 can reliably be used on TMAs [28]. We observed good concordance between Ki67 scores on whole slides versus TMA. Furthermore, the inter-observer variability for Ki67 scoring within our laboratory was very low, indicated by a high kappa value. A discrepancy between Ki67 and mitotic count has previously been described. Jalava et al. [29] observed that mitotic count was a better predictor for prognosis than Ki67. In their study, patients with low Ki67 levels and high mitotic count had an unfavorable prognosis, similar to those patients whose tumor expressed both high Ki67 as well as high mitotic count. Considering that Ki67 levels are low in the G1 and S phases and rise to their peak level in mitosis [9], a biological explanation for this observed discrepancy remains unclear. In addition, although the Ki67 protein seems to have an important role in cell division, its exact function has not been fully elucidated [9].

In contrast to the currently recommended treatment duration of at least 5 years, the duration of tamoxifen treatment in our series was only 1–3 years. We cannot exclude that prolonged tamoxifen treatment would have been beneficial for patients with high mitotic count. However, these patients were at particularly high risk of early recurrences as indicated by the Schoenfeld residuals. Therefore, a potential risk reduction of tamoxifen would be most pronounced in the first few years after diagnosis in patients with tumors with a high mitotic count. Time dependent hazard ratios, similar to our observation for mitotic count, have previously been described by Hilsenbeck et al. [30]. It would be valuable to test the predictive value of mitotic count in a trial of 5 years tamoxifen versus nil, like the NSABP-14 trial [31].

Similar to previous results in premenopausal patients [15] we observed a significant interaction between *CCND1* copy number as assessed with probe 2 and tamoxifen in postmenopausal patients. Patients whose tumor expressed a high log2 copy number ratio of *CCND1* as assessed with probe 2 did not benefit from tamoxifen. We did not observe an association between *CCND1* log2 copy number ratio and Cyclin D1 protein expression. This is in agreement with results observed by Bostner et al. [16] and can be explained by post-transcriptional regulation of nuclear Cyclin D1 [32]. In line with our findings regarding *CCND1* probeset 1 that is close to the probeset Bostner et al. used (see Additional file 2: Figure S2), Bostner et al. did not observe a significant interaction between *CCND1* amplification and tamoxifen [16]. They did however observe a significant interaction with *PAK1* amplification [16]. Of note, the patient numbers in their study (*N* = 153) were much lower than in our series (*N* = 450). As previously suggested [33], this hints to the presence of several independent amplification cores instead of involvement of a single large amplicon. This may also explain why we did not observe a strong correlation between the different *CCND1* probes and *EMSY* probes.

Clinically relevant would be to know what the optimal adjuvant treatment in patients would be with either high mitotic count or amplification of *CCND1*. Considering the reduced benefit from tamoxifen only, one could argue that chemotherapy should be added in these patients. Very recently, cell cycle inhibitors have been shown to be beneficial in metastatic breast cancer patients when added to endocrine therapy, both in CCND1 amplified tumors as well as in unselected patients [34]. A potential role of these new drugs in the adjuvant setting needs to be explored. Our data suggest that patients, whose tumors express high mitotic count or *CCND1* amplification, would be suitable candidates for such therapies.

Several multigene tests, such as PAM50-based risk of recurrence (ROR), 21-gene recurrence score, IHC4 score, Breast Cancer Index, and Endopredict Clinical Treatment Score have been investigated for predicting outcome after endocrine therapy (± chemotherapy) in ER-positive, HER2-negative patients [35, 36]. All six multigene tests added independent prognostic information to the so-called Clinical Treatment Score, based on tumor size, nodal status, histological grade, age and treatment received (tamoxifen or anastrozole) in node-negative, postmenopausal patients, who had not received chemotherapy [35]. However, whether these tests have tamoxifen treatment predictive value is unclear [37]. The 21-gene recurrence score has been tested for a treatment-by-biomarker interaction in a subset of the NSABP B-14 trial and a trend was observed [8]. When the 16 cancer-related genes of the test were analyzed separately, the *ESR1* transcript level was highly predictive of adjuvant tamoxifen benefit, while the *MKI67* transcript level, encoding the Ki67 protein, was not [8]. The latter result is in line with our findings. Recently, an ultralow risk cut-off for the 71-gene signature suggested that this test has both prognostic as well as predictive value regarding adjuvant tamoxifen benefit in N0 postmenopausal patients [38]. While these multigene tests seem clinically valuable, these tests are relatively expensive, and often not readily available. Particularly in those countries where access to these tests is not possible, simply testing the mitotic count may already give important additional information to decide about adjuvant therapies. Furthermore, in those instances where multigene tests return intermediate risk results, a low mitotic count may indicate substantial benefit from tamoxifen that may help guiding decisions on adjuvant chemotherapy.

## Conclusions

In conclusion we have shown that postmenopausal patients with high Ki67 counts do benefit from adjuvant tamoxifen. *CCND1* may be predictive for reduced efficacy of adjuvant tamoxifen. Moreover, mitotic count, a commonly assessed prognostic factor in breast cancer, might be an additional factor that can be used to predict the likelihood to derive benefit from adjuvant tamoxifen only. These findings need confirmation in at least one independent study before implementation in the clinic [37].

### Additional files

Additional file 1 **Table S1:** Distribution of clinico-pathological variables between patients with sufficient tumor material for biomarker analysis and the total group of patients who entered the study patients with sufficient tumor material. **Table S2:** Inter-observer variability for Ki67 and cyclin D1 immunohistochemistry scores antibody scoring system comparable cores. **Table S3:** Specifications of REMARK recommendations. **Table S4:** Multivariate Cox proportional hazard model of recurrence free interval (RFI) including mitotic count and treatment interaction, follow up truncated at 6 years. **Table S5:** Multivariate Cox proportional hazard model of recurrence free interval (RFI) including mitotic count and treatment interaction, follow up truncated at 6 years in HER2 negative patients. **Table S6:** Multivariate Cox proportional hazard model of recurrence free interval (RFI) including CCND1 copy number ratio and treatment interaction. **Table S7a:** Interaction tests between tamoxifen and EMSY probe sets analyzed as continuous. **Table S7b:** Interaction tests between tamoxifen and EMSY probe sets analyzed as binary factor. (PDF 368 kb)

Additional file 2 **Figure S1** Location of the different CCND1 and EMSY probe sets in the genome. In addition the CCND1 and PAK1 probes used for PCR by Bostner are depicted. The UCSC Genome Browser was used to visualize the loci of interest in hg19 coordinates.**Figure S2** A mixed effects regression of the log2-transformed reference sample estimates were modeled with reference probe-set, batch and their interaction as a fixed effect and sample as a random effect. Presented is a bar plot is of the variance in the batch estimates per probe-set. **Figure S3** Data flow of patients entering the study, the reason of exclusion and finally analyzed for the specific markers.**Figure S4** differences between Ki67 score on whole tissue slide and maximum score from 3 corresponding cores on TMA from tumors of a random series of 55 patients (comparable scores were available for 54 patients, since the staining on whole tissue slide failed for 1 tumor). **Figure S5** Distribution of scores for mitosis markers: CCND1 probe set 1, CCND1 probe set 2, immunohistochemistry markers Ki67 and Cyclin D1, mitotic count per 2 mm2 and the square root transformed mitotic count per 2 mm2. **Figure S6** Schoenfeld residuals for mitotic count (high (≥ 8 mitosis/2 mm2) versus low (< 8 mitosis/2 mm2)) over years in the entire cohort of 557 ER α positive patients for whom mitotic count could be assessed. Recurrence free interval survival was stratified by nodal status. (DOC 731 kb)

## Appendix

MLPA reaction protocol. We first denatured a total of 50 ng of template DNA dissolved in TE in a volume of 2.5 μl in a thermocycler at 98 °C for 5 min before allowing to cool to 25 °C. We then hybridized the denatured DNA with 1.5 μl of MLPA buffer and 1.5 μl of P078-B1 probe mix at 95 °C for 1 min, then 60 °C for 18 h. A volume of 4 μl of the hybridization reaction was then added to 1.5 μl of Ligase-65 buffer A, 1.5 μl of Ligase-65 buffer B, 12.5 μl of water and 0.5 μl of Ligase-65 and ligated at 15 °C for 15 min, then heat inactivated at 98 °C for 5 min. Next, the ligation reaction was diluted 1:2 with water and 1 μl was added to 1 μl of Polymerase mix and 3 μl of SALSA PCR buffer mix. Polymerase mix contains 0.2 μl of SALSA PCR primers, 0.2 μl of SALSA Enzyme Dilution Buffer, 0.55 μl water and 0.05 μl of SALSA polymerase. SALSA PCR buffer mix contains 0.4 μl of SALSA PCR buffer and 2.6 μl of water. While at 60 °C, the polymerase mix was added to the ligation reaction and PCR buffer mixture. PCR conditions for the reaction began immediately with 30 cycles of 95 °C denaturation for 30 s, hybridization for 30 s at 60 °C and an extension of 72 °C for 60 s. There was an additional extension of 20 min at 72 °C. A volume of 2 μl of PCR product was then added to 9.8 μl of HiDi Formamide (Roche) and 0.2 μl of ROX 500 standard (Invitrogen). Fragment separation was carried out on the ABI-3730 according to manufacturer’s suggestions. We analyzed the series in 15 batches, with each experiment containing duplicate reference DNA samples. Reference DNA was a pool of 8 normal individuals sheared to simulate DNA.

## Abbreviations

CCND1: Gene encodes the cyclin D1 protein
ERα: Estrogen receptor alpha
FFPE: Formalin-fixed paraffin embedded
HER2: Human epidermal growth factor receptor 2
HR: Hazard ratio
IHC: Immunohistochemistry
MPLA: Multiplex ligation-dependent probe amplification
PgR: Progesterone receptor
RFI: Recurrence free interval
TMA: Tissue micro-arrays
