# Supplementary material for: Mitotic count can predict tamoxifen benefit in postmenopausal breast cancer patients while Ki67 score cannot
Source: BMC Cancer. 2018 Jul 24;18:761. doi: 10.1186/s12885-018-4516-1 (PMC6057037; doi:10.1186/s12885-018-4516-1)
Supplement: Supplementary file 1 — Table S1: Distribution of clinico-pathological variables between patients with sufficient tumor material for biomarker analysis and the total group of patients who entered the study patients with sufficient tumor material. Table S2: Inter-observer variability for Ki67 and cyclin D1 immunohistochemistry scores antibody scoring system comparable cores. Table S3: Specifications of REMARK recommendations. Table S4: Multivariate Cox proportional hazard model of recurrence free interval (RFI) including mitotic count and treatment interaction, follow up truncated at 6 years. Table S5: Multivariate Cox proportional hazard model of recurrence free interval (RFI) including mitotic count and treatment interaction, follow up truncated at 6 years in HER2 negative patients. Table S6: Multivariate Cox proportional hazard model of recurrence free interval (RFI) including CCND1 copy number ratio and treatment interaction. Table S7a: Interaction tests between tamoxifen and EMSY probe sets analyzed as continuous. Table S7b: Interaction tests between tamoxifen and EMSY probe sets analyzed as binary factor. (PDF 368 kb) [file 12885_2018_4516_MOESM1_ESM.pdf]

Table S1 Distribution of clinico-pathological variables between patients with sufficient tumor material for biomarker analysis and the total group of patients who entered the study

		**patients with sufficient tumor material**	**total study population**	
		N (%)	*N* (%)	
	total	739 (100)	1662 (100)	*p* value[a]
**age**	<65	378 (51)	869 (52)	0.59
	≥ 65	361 (49)	793 (48)	
**lymph node**	neg	393 53)	901 (54)	0.61
	pos	346 (47)	761 (46)	
**T stage**	T 1-2	659 (89)	1482 (89)	0.99
	T 3-4	80 (11)	180 (11)	
**ER status[b]**	pos	468 (77)	1014 (77)	0.87
	neg	142 (23)	311 (23)	
**PR status[b]**	pos	224 (57)	513 (60)	0.31
	neg	168 (43)	346 (40)	

[a]*Chi-Square test*
[b]*As defined with ligand binding assay. After revision of recollected tumors, a total of 563 were ER positive as assessed with immunohistochemistry*

Table S2: Inter-observer variability for Ki67 and cyclin D1 immunohistochemistry scores

**antibody**	**scoring system**	**comparable cores from *N* patients**	**cutoff for binary score**	**Kappa for binary score**
ki67	proportion of positive nuclei	74	5% or more versus below 5%	0.89
Cyclin D	proportion of positive nuclei	59	above median versus below median	0.55

Supplementary table 3. Specifications of REMARK recommendations

**Introduction**	
Marker	Cell proliferation markers: mitosis count, Ki67, cyclin D1 protein expression and *CCND1* copy number variation.
Objectives	To evaluate the predictive capacity of the different cell proliferation markers for benefit from adjuvant tamoxifen in postmenopausal breast cancer patients.
Hypothesis	High expression of cell proliferation markers would result in decreased benefit from adjuvant tamoxifen in postmenopausal breast cancer patients. .

**Methods (1)**	**Patients**
Characteristics	From 1982 until 1994 a randomized clinical trial was conducted in the Netherlands, studying the benefit of adjuvant tamoxifen (IKA-trial) in postmenopausal breast cancer patients.
Inclusion criteria	In the original study, 1662 breast cancer patients were included who were post-menopausal, less than 76 years of age and had a $T_{1–4}$, $N_{0–3,}$ M_0 breast tumor. We have traced tissue blocks of participating patients and recollected sufficient tumor material of 739 patients, who did not differ in prognostic factors from the total group (Table S1). After revision of estrogen receptor α (ERα) status as assessed with immunohistochemistry (IHC), a total of 563 ERα positive (≥10%) tumors were used for subsequent analysis.
Exclusion criteria	Mastitis or palpable supra- or infraclavicular lymph nodes
Treatment	Patients were randomized in a 2:1 distribution between 1 year tamoxifen (30 mg per day) versus no adjuvant therapy. After 1 year a second randomization was performed to receive another 2 years of tamoxifen or to stop further treatment. From 1989, based on two interim analyses showing a significant improvement in recurrence free survival in lymph node positive patients, these node positive patients were all allocated to the tamoxifen treatment arm (ie skipped the first randomization).

**Methods (2)**	**Specimen characteristics**
Material used	Formalin-fixed paraffin-embedded (FFPE) breast tumor tissue of the primary tumor. DNA was isolated from FFPE material.
Preservation/storage	Formalin fixation and paraffin embedding. Storage at room temperature. Tumor DNA was stored at 4ºC.

Methods (3)	Assay methods
Assay	Mitosis count was assessed (PvD) on an HE slide per 2 mm^2 as described before. Immunohistochemistry for Ki67 was performed using the monoclonal mouse anti-human Ki67 antigen, clone MIB-1 (DAKO) and a standard staining protocol on the Ventana Benchmark® Ultra system. Cyclin D1 protein expression was assessed using the cyclin D1 / Bcl-1(SP4) antibody (Neomarkers) and a standard staining protocol on the Labvision system. *CCND1* gene copy number variation was assessed with multiplex ligation-dependent probe amplification-based copy number analysis (MLPA). The P078-B1 Breast tumor probe-mix (MRC Holland, Amsterdam, the Nethetlands) was used, which contains probe sets for several genes that frequently show copy number changes in breast tumors
Protocol	Staining protocols can be downloaded from our website: https://www.nki.nl/topmenu/protocols-sabine-linn/ . We carried out MLPA reactions according to the manufacturer's protocol for 2010 (appendix 1)
Control experiments	For MLPA analysis, Reference DNA was a pool of 8 normal individuals sheared to simulate FFPE DNA. Normal surrounding tissue was taken along as control for immunohistochemistry
Reproducibility	For each immunohistochemical staining, one of the TMAs was quantified independently in a blinded manner by a second observer to calculate inter-observer variability. The inter-observer variability analyzed using the (weighted) Cohen's kappa coefficient is depicted in Table S1
Quantification	Quantification of immunohistochemical staining was performed as described in the method section for immunohistochemistry. For MLPA analysis, normalization of the signals and calculation of copy number ratio are described in section methods.
Blinding	Scoring of the immunohistochemical stainings was done without knowledge regarding both the recurrence-free-interval survival as well as the treatment arm at the time of scoring.

Methods (4)	Study design I
Case selection	A randomized controlled trial. The translational study presented her was performed retrospectively. The median duration of follow-up for patients without a recurrence event was 7.8 years. Patient records were re-evaluated for recurrence until 2000.
Clinical endpoints	The improvement of recurrence free interval (RFI) with tamoxifen versus nil was assessed according to the different proliferation markers as mentioned below. RFI included local, regional, distant recurrences and breast cancer-specific death, but not contra-lateral breast cancer, as the primary event.
Variables examined or considered	Multivariate Cox models included age (≥ 65 versus < 65), grade (grade 3 versus grade 1-2), tumor size (T3-4 versus T1-T2), HER2 status (positive versus negative) and progesterone status (positive versus negative) as covariates.
Rational for sample size	The sample size of the translational study is based on the amount of available tumor blocks containing invasive, ERα positive tumor cells, that could be recollected and a power calculation based on events in this group assuring that meaningful results could be deduced.

Methods (5)	Statistical analysis
Statistical methods and variable selection procedure	Recurrence free interval was defined as the time from the date of first randomization until the occurrence of a local, regional or distant recurrence or breast cancer specific death. A secondary contra-lateral breast tumor was not considered as an event and these patients were censored at the date of this occurrence. Survival curves were constructed using the Kaplan-Meier method and compared using the log-rank test. All p-values are based on a two-sided test. All calculations were made with Statistical Package for the Social Sciences (SPSS) 15.0 Inc., IL, USA.
Missing data	Cases with a missing value for one of the variables were excluded from the multivariate analysis.
Marker handling in analysis	To test whether the benefit from tamoxifen was dependent on proliferation markers, unadjusted and adjusted interaction tests were performed using Cox proportional hazard regression. As continuous linear variables were tested: Ki67 score, mitotic count (square root transformed), cyclin D1 and CCND1 and EMSY log 2 copy number ratio (probe sets 1 and probe sets 2 were tested separately). In addition, we tested Ki67, mitotic count and cyclin D1 as binary factor using the median as cutoff. For analysis of CCND1 and EMSY log2 copy number ratio as binary factor, 0 was defined as cutoff.

Results (1)	Data
Flow of patients	See Figure S3 for description of patients excluded for this translational study. See Table S1 for characteristics of total study patients versus the 739 patients with sufficient tumor material included in TMA .
Characteristics	See Table 2.

Results (2)	Analysis and presentation
Relation to standard prognostic variables	See Table 2.
Univariate analysis	See Figures 2 and 3.
Multivariate analysis	See Tables 1 and S4-7.

Discussion	
Interpretation, limitations and implication	See discussion section

Table S4: Multivariate Cox proportional hazard model of recurrence free interval (RFI) including mitotic count and treatment interaction, follow up truncated at 6 years

Variable	*N*(events)	Hazard Ratio*	95% CI	*p*-value
**Age**				
< 65	246 (43)	1.00		
≥ 65	269 (49)	1.02	0.67-1.55	0.92
**p T-stage**				
T1 or T2	458 (75)	1.00		
T3 or T4	57 (17)	1.46	0.84 – 2.52	0.18
**Histologic grade**				
I-II	337 (43)	1.00		
III	178 (49)	2.04	1.05-3.95	0.04
**Progesterone receptor**				
negative	248 (46)	1.00		
positive	267 (46)	0.95	0.63– 1.45	0.82
**HER2 status**				
negative	474 (81)	1.00		
positive	41 (11)	1.08	0.56– 2.11	0.82
**Mitotic count**				
Low (< 8 / $2mm^{2)}$	253 (33)	1.00		
High (≥ 8 / $2mm^{2)}$	262 (59)	0.47	0.20-1.16	0.10
**Treatment**				
Low mitosis and control	59 (14)	1.00		
Low mitosis and tamoxifen	194 (19)	0.24	0.12-0.49	<0.0001
High mitosis and control	62 (15)	1.00		
High mitosis and tamoxifen	200 (44)	0.64	0.35-1.17	0.14
Interaction mitosis and X treatment				0.03

*stratified for nodal status

Analysis based on 515 cases with 92 events

Table S5: Multivariate Cox proportional hazard model of recurrence free interval (RFI) including mitotic count and treatment interaction, follow up truncated at 6 years in HER2 negative patients

		[redacted]		
**Variable**	***N*(events)**	**Hazard Ratio***	**95% CI**	***p*-value**
**Age**				
< 65	228 (38)	1.00		
≥ 65	246 (43)	1.02	0.65-1.58	0.95
**p T-stage**				
T1 or T2	424 (67)	1.00		
T3 or T4	50 (14)	1.45	0.80 – 2.63	0.22
**Histologic grade**				
I-II	328 (42)	1.00		
III	146 (39)	2.19	1.09-4.42	0.03
**Progesterone receptor**				
negative	218 (37)	1.00		
positive	256 (44)	1.03	0.66– 1.62	0.88
**Mitotic count**				
Low (< 8 / 2mm^{2})	250 (33)	1.00		
High (≥ 8 / 2mm^{2})	224 (48)	0.48	0.19-1.23	0.13
**Treatment**				
Low mitosis and control	59 (14)	1.00		
Low mitosis and tamoxifen	191 (19)	0.24	0.12-0.49	<0.0001
High mitosis and control	56 (14)	1.00		
High mitosis and tamoxifen	168 (34)	0.56	0.30-1.07	0.08
Interaction mitosis and X treatment				0.07

*stratified for nodal status

Analysis based on 474 cases with 81 events

Table S6: Multivariate Cox proportional hazard model of recurrence free interval (RFI) including **C**CND1 copy number ratio and treatment interaction

Variable	*N* (events)	Hazard Ratio*	95% CI	*p*-value
**Age**				
< 65	210 (52)	1.00		
≥ 65	229 (50)	1.00	0.67-1.49	1.00
**p T-stage**				
T1 or T2	391 (85)	1.00		
T3 or T4	48 (17)	1.52	0.88 – 2.62	0.13
**Histologic grade**				
I-II	284 (53)	1.00		
III	155 (49)	1.58	1.03-2.40	0.03
**Progesterone receptor**				
negative	216 (45)	1.00		
positive	223 (57)	1.23	0.82– 1.85	0.31
**HER2 status**				
negative	404 (92)	1.00		
positive	35 (10)	0.96	0.48-1.90	0.90
**CCND1 copy number ratio**				
Low (< 0)	404 (92)	1.00		
High (> 0)	35 (10)	0.55	0.26-1.15	0.11
**Treatment**				
CCND1 low and control	39 (15)	1.00		
CCND1 low and tamoxifen	151 (28)	0.32	0.16-0.61	0.001
CCND1 high and control	69 (14)	1.00		
CCND1 high and tamoxifen	180 (45)	0.81	0.44-1.52	0.52
Interaction CCND1 X treatment				0.04

*stratified for nodal status

Analysis based on 439 cases with 102 events

Table S7a: Interaction tests between tamoxifen and EMSY probe sets analyzed as continuous linear variables

**variable**	**variable values**	***N* (events)**	**Interaction *p*-value**
**EMSY probeset 1 log2 copy number ratio**	-1.46 to 3.98	454 (103)	0.10
**EMSY probeset 2 log2 copy number ratio**	-1.79 to 2.88	454 (104)	0.98

Table S7b: Interaction tests between tamoxifen and EMSY probe sets analyzed as binary factor

	**log2 copy nr ratio**	**HR (95% CI) for tamoxifen versus control**	***p*-value for interaction**
**EMSY probeset 1**	< 0	0.62 (0.25-1.55)	0.67
	> 0	0.49 (0.29-0.83)	
**EMSY probeset 2**	< 0	0.65 (0.30-1.43)	0.45
	> 0	0.45 (0.26-0.79)	
