# Supplementary material for: Mitotic count can predict tamoxifen benefit in postmenopausal breast cancer patients while Ki67 score cannot
Source: BMC Cancer. 2018 Jul 24;18:761. doi: 10.1186/s12885-018-4516-1 (PMC6057037; doi:10.1186/s12885-018-4516-1)
Supplement: Supplementary file 2 — Figure S1 Location of the different CCND1 and EMSY probe sets in the genome. In addition the CCND1 and PAK1 probes used for PCR by Bostner are depicted. The UCSC Genome Browser was used to visualize the loci of interest in hg19 coordinates.Figure S2 A mixed effects regression of the log2-transformed reference sample estimates were modeled with reference probe-set, batch and their interaction as a fixed effect and sample as a random effect. Presented is a bar plot is of the variance in the batch estimates per probe-set. Figure S3 Data flow of patients entering the study, the reason of exclusion and finally analyzed for the specific markers.Figure S4 differences between Ki67 score on whole tissue slide and maximum score from 3 corresponding cores on TMA from tumors of a random series of 55 patients (comparable scores were available for 54 patients, since the staining on whole tissue slide failed for 1 tumor). Figure S5 Distribution of scores for mitosis markers: CCND1 probe set 1, CCND1 probe set 2, immunohistochemistry markers Ki67 and Cyclin D1, mitotic count per 2 mm2 and the square root transformed mitotic count per 2 mm2. Figure S6 Schoenfeld residuals for mitotic count (high (≥ 8 mitosis/2 mm2) versus low (< 8 mitosis/2 mm2)) over years in the entire cohort of 557 ER α positive patients for whom mitotic count could be assessed. Recurrence free interval survival was stratified by nodal status. (DOC 731 kb) [file 12885_2018_4516_MOESM2_ESM.doc]

**Supplementary figure1:** Location of the different CCND1 and EMSY probe sets in the genome. In addition the CCND1 and PAK1 probes used for PCR by Bostner are depicted. The UCSC Genome Browser was used to visualize the loci of interest in hg19 coordinates.

**Supplementary figure 2:** A mixed effects regression of the log2-transformed reference sample estimates were modeled with reference probe-set, batch and their interaction as a fixed effect and sample as a random effect. Presented is a bar plot is of the variance in the batch estimates per probe-set.

**Supplementary figure 3**: Data flow of patients entering the study, the reason of exclusion and finally analyzed for the specific markers.

**Supplementary figure 4**: differences between Ki67 score on whole tissue slide and maximum score from 3 corresponding cores on TMA from tumors of a random series of 55 patients (comparable scores were available for 54 patients, since the staining on whole tissue slide failed for 1 tumor)

**Supplementary figure 5**: Distribution of scores for mitosis markers: CCND1 probe set 1, CCND1 probe set 2, immunohistochemistry markers Ki67 and Cyclin D1, mitotic count per 2mm^2^ and the square root transformed mitotic count per 2mm^2^.

**Supplementary figure 6**: Schoenfeld residuals for mitotic count (high (≥ 8 mitosis/2mm^2^ ) versus low (< 8 mitosis/2mm^2^ )) over years in the entire cohort of 557 ER α positive patients for whom mitotic count could be assessed. Recurrence free interval survival was stratified by nodal status.
